# NPInter v5.0: ncRNA interaction database in a new era

**DOI:** 10.1093/nar/gkac1002

**Published:** 2022-11-14

**Authors:** Yu Zheng, Huaxia Luo, Xueyi Teng, Xinpei Hao, Xiaoyu Yan, Yiheng Tang, Wanyu Zhang, Yuanxin Wang, Peng Zhang, Yanyan Li, Yi Zhao, Runsheng Chen, Shunmin He

**Affiliations:** Key Laboratory of RNA Biology, Center for Big Data Research in Health, Institute of Biophysics, Chinese Academy of Sciences, Beijing 100101, China; College of Life Sciences, University of Chinese Academy of Sciences, Beijing 100049, China; Key Laboratory of RNA Biology, Center for Big Data Research in Health, Institute of Biophysics, Chinese Academy of Sciences, Beijing 100101, China; Key Laboratory of RNA Biology, Center for Big Data Research in Health, Institute of Biophysics, Chinese Academy of Sciences, Beijing 100101, China; Key Laboratory of RNA Biology, Center for Big Data Research in Health, Institute of Biophysics, Chinese Academy of Sciences, Beijing 100101, China; University of Chinese Academy of Sciences, Beijing 100049, China; Key Laboratory of RNA Biology, Center for Big Data Research in Health, Institute of Biophysics, Chinese Academy of Sciences, Beijing 100101, China; University of Chinese Academy of Sciences, Beijing 100049, China; Key Laboratory of RNA Biology, Center for Big Data Research in Health, Institute of Biophysics, Chinese Academy of Sciences, Beijing 100101, China; Key Laboratory of RNA Biology, Center for Big Data Research in Health, Institute of Biophysics, Chinese Academy of Sciences, Beijing 100101, China; Key Laboratory of RNA Biology, Center for Big Data Research in Health, Institute of Biophysics, Chinese Academy of Sciences, Beijing 100101, China; University of Chinese Academy of Sciences, Beijing 100049, China; Key Laboratory of RNA Biology, Center for Big Data Research in Health, Institute of Biophysics, Chinese Academy of Sciences, Beijing 100101, China; Key Laboratory of RNA Biology, Center for Big Data Research in Health, Institute of Biophysics, Chinese Academy of Sciences, Beijing 100101, China; Bioinformatics Research Group, Key Laboratory of Intelligent Information Processing, Advanced Computing Research Center, Institute of Computing Technology, Chinese Academy of Sciences, Beijing 100190, China; Key Laboratory of RNA Biology, Center for Big Data Research in Health, Institute of Biophysics, Chinese Academy of Sciences, Beijing 100101, China; Key Laboratory of RNA Biology, Center for Big Data Research in Health, Institute of Biophysics, Chinese Academy of Sciences, Beijing 100101, China; University of Chinese Academy of Sciences, Beijing 100049, China

## Abstract

Noncoding RNAs (ncRNAs) play key regulatory roles in biological processes by interacting with other biomolecules. With the development of high-throughput sequencing and experimental technologies, extensive ncRNA interactions have been accumulated. Therefore, we updated the NPInter database to a fifth version to document these interactions. ncRNA interaction entries were doubled from 1 100 618 to 2 596 695 by manual literature mining and high-throughput data processing. We integrated global RNA–DNA interactions from iMARGI, ChAR-seq and GRID-seq, greatly expanding the number of RNA–DNA interactions (from 888 915 to 8 329 382). In addition, we collected different types of RNA interaction between SARS-CoV-2 virus and its host from recently published studies. Long noncoding RNA (lncRNA) expression specificity in different cell types from tumor single cell RNA-seq (scRNA-seq) data were also integrated to provide a cell-type level view of interactions. A new module named RBP was built to display the interactions of RNA-binding proteins with annotations of localization, binding domains and functions. In conclusion, NPInter v5.0 (http://bigdata.ibp.ac.cn/npinter5/) provides informative and valuable ncRNA interactions for biological researchers.

## INTRODUCTION

The ENCODE project has revealed that up to 80% of the human genome is transcribed but <2% of the genome encodes proteins, with the vast majority of RNA transcripts being ncRNAs (1). ncRNAs can regulate cell physiology and shape cellular functions in the whole biological regulatory network through interactions with other biomolecules, such as proteins, mRNAs and the genome (2). For example, microRNAs (miRNAs), well-known small single-stranded ncRNAs, usually bind to the 3′-untranslated region (UTR) of target mRNAs and drive post-transcriptional gene regulation (3). The lncRNA, *XIST*, can regulate dosage compensation by interacting with X chromosome in the female embryo (4). The study of ncRNA interaction is important in ncRNA function research.

Given the importance of ncRNA interactions in biological regulation, we constructed and released the first version of NPInter in 2006 to gather and arrange ncRNA interactions from experimental validation and high-throughput data (5). In the subsequent 15 years, we have updated and improved the database in three versions (6–8). In recent years, many experimental and high-throughput technologies have been developed and widely applied to identify RNA interactions, such as PARIS (9), SPLASH (10), LIGR-seq (11), CLIP-seq (12), ChIRP-seq (13) and MARGI (14). ncRNA interactome data has expanded extraordinarily and covers numerous organisms and tissues. We therefore updated NPInter to a fifth version to integrate these expanding resources and to provide comprehensive functional annotations to help ncRNA researchers look into the whole transcriptomic regulatory networks. In addition to a doubling of entries compared with the last version, we have also added global RNA–DNA interactions detected by iMARGI (15), ChAR-seq (16) and GRID-seq (17), which greatly expand the number of interactions between ncRNAs and chromosomes. To contribute to COVID-19 research, we also collected different types of RNA interaction between SARS-CoV-2 virus and its host from recently published studies. In addition, lncRNA expression specificity in different cell types from tumor scRNA-seq data were integrated into this version to provide a cell-type level view of interactions. Based on user feedback, we built the RBP module, which deposits RNA-binding proteins (RBPs) with annotations of localization, binding domains and functions. All interaction data can be freely downloaded from the download page.

## DATA COLLECTION AND ANNOTATION

**Figure 1.**
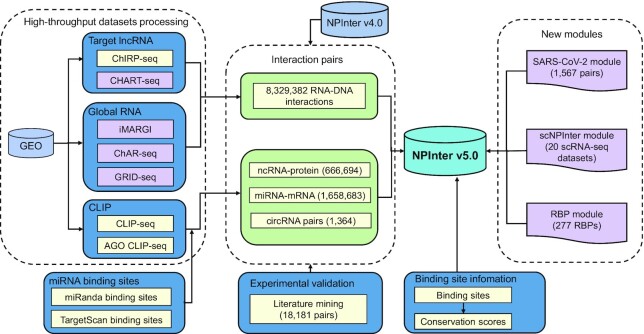
The overall workflow of NPInter v5.0 data integration. ncRNA interactions were obtained by literature mining and high-throughput datasets processing. ChIRP-seq, CLIP-seq and AGO CLIP-seq datasets were processed to obtain RNA–DNA, lncRNA–protein and miRNA–mRNA interactions, respectively. CHART-seq, iMARGI, ChAR-seq and GRID-seq data were processed to obtain RNA–DNA interactions, which are newly added in this version of NPInter. Purple frames (SARS-CoV-2, scNPInter, RBP) indicate new modules integrated into NPInter v5.0.

In NPInter v5.0, new RNA interactions from manual literature mining and high-throughput datasets were integrated with NPInter v4.0 interactions (8). Interactions from different sources were added to the database with redundancy removed. Biomolecules were then allocated with standard IDs from reference databases according to their categories. The overall workflow of data integration is shown in Figure 1.

### Interactions curated from literature mining

To collect experimentally validated interactions between ncRNAs and biomolecules (proteins, RNAs and DNAs), we manually curated interaction pairs from public literature documented in PubMed. Over ten thousand related papers published between May 2019 and March 2022 were collected. After manual and stringent curation, we obtained 8587 interactions with experimental evidence from 5143 articles. The keywords for the PubMed literature search are listed in the Supplementary Material. In addition, we specifically collected SARS-CoV-2 related RNA interactions from literature published since 2020.

### RNA–DNA interactions from ChIRP-seq and CHART-seq data

To collect RNA–DNA interactions from high-throughput datasets, we downloaded and processed 156 raw SRA ChIRP-seq datasets (13) and 117 CHART-seq datasets (18). CHART-seq data was newly added in this version. Raw sequence reads were mapped to the reference genome (hg19, mm9) using bwa (19), and then MACS2 (20) was employed to call peaks. Genome binding sites of each ncRNA were annotated with GENCODE annotation (such as intron, exon and UTR) (21), and sites in intergenic regions were annotated with the nearest gene. To display the genome-wide distribution of RNA–DNA interactions, we calculated binding peak numbers for each ncRNA across 1 Mb window and visualized it using BioCircos.js (22).

### Global RNA–DNA interactions

For global RNA–DNA interactions, we collected data from techniques including iMARGI, ChAR-seq and GRID-seq, which globally localize chromatin-associated ncRNAs, much more efficiently than the single RNA-targeting methods of ChIRP-seq and CHART-seq. ncRNA molecules were extracted and annotated with disease information from raw datasets of these three technologies. Information of experimental technologies for each interaction was retained. We compared the RNA–DNA binding sites with ncRNA annotations deposited in NONCODE v6 database (23) and allocated NONCODE IDs to these ncRNAs. Annotation and distribution of genome-wide binding sites on chromosomes were processed as described in the previous section.

### RNA–protein interactions from CLIP-seq datasets

To gather RNA–protein interactions, we preprocessed 130 GSE datasets with 308 protein targets from the Gene Expression Omnibus (GEO) (24) database, covering human and mouse interactome data. STAR (25) was used to map raw sequences to reference genomes (hg19 for human, mm9 for mouse), and then piranha software (26) was applied to call peaks interacting with specific RNA-binding proteins. To ensure the quality of calling peaks, *P* value threshold was set to 0.001. RNA–protein binding sites were also compared with ncRNA annotations in NONCODE v6 database and ncRNAs were allocated NONCODE IDs. Evolutionary conservation score of binding site was calculated for each interaction using PhastCons (27).

### miRNA targets from Ago-CLIP datasets

We processed Argonaute targeted CLIP-seq (AGO-CLIP) datasets based on the method described in NPInter v4.0 (8). The *P* value threshold was also set to 0.001 in software piranha for good quality of peaks. BEDOPS (28) was used to extract overlaps of miRNA binding sites deposited in miRanda (29) and TargetScan (30) and miRNA targeting peaks derived from Ago-CLIP datasets. RNAs with such overlapping binding sites are predicted to be credible miRNA interaction partners. Moreover, we also assigned standard molecule IDs to these molecules.

### Re-annotation, removal of redundancy and integration

We re-annotated biomolecules in NPInter with standard IDs from reference databases. Specifically, lncRNAs, miRNAs, circRNAs, mRNAs and proteins were annotated with NONCODE IDs (23), miRBase IDs (31), circBase IDs (32), Ensembl IDs (33) and UniProt IDs (34) respectively. Additionally, an Ensembl ID and a RefSeq ID (35) for each molecule were provided if available, which facilitates biomolecule search. We integrated tissues, experiments, data sources and references for a large number of interaction pairs collected from different sources and allocated new interaction IDs for these interactions after eliminating repetitive entries.

### lncRNA annotation from scRNA-seq data

In NPInter v5.0, we newly integrated lncRNA information with cell type and cell state from cancer scRNA-seq datasets. Based on cancer types in TCGA (https://portal.gdc.cancer.gov/), we collected information of over 50 tumor scRNA-seq datasets from the literature and downloaded related data sources from GEO. After screening, 20 scRNA-seq datasets representing 20 cancer types were selected with complete cell type annotation and available expression count matrix. The Seurat (36) package was used to accomplish a standard pre-processing workflow, which included normalization, clustering and non-linear dimensional reduction for raw expressing count matrix. Then, we manually assigned cell type to each cluster according to marker genes extracted from the original paper and identified specifically expressed lncRNAs in each cell type. Interactions related to these cell type-specific lncRNAs were collected and organized for display in the scNPInter module on the website.

## DATABASE CONTENT AND STRUCTURE

### Interactions and annotated information

The volume of data is greatly expanded in NPInter v5.0. We doubled the number of interactions (without counting RNA–DNA bindings) from 1 100 618 in NPInter v4.0 to 2 596 695 interactions in NPInter v5.0 covering 60 species by literature mining and high-throughput data processing (Table 1). Interactions were attributed to different categories according to the specific types of biomolecules involved (such as ‘lncRNA–protein’, ‘miRNA–mRNA’, etc) (Table 2). In particular, we collected 8,586 experimentally validated interactions via literature mining from 5143 articles published after 2019, a larger number than in NPInter v4.0 (1221 papers). The integration of interactions from global RNA–DNA interactome technologies caused the number of RNA–DNA interaction pairs to surge from 888 915 to 8 329 382 (Table 3).

**Table 1.** Statistics of interactions from different data sources

Data sources	Interactions
Literature mining	18 181
CLIP-seq data	661 938
miRNA–mRNA interactions from Ago CLIP-seq data	1 787 679
Other database^a^	128 897

^a^RNA interactions from RISE database.

**Table 2.** Statistics of different categories of interactions

Data sources	Interactions
lncRNA–protein	662 956
lncRNA-miRNA	155 140
lncRNA–mRNA	17 451
miRNA–protein	3656
miRNA–mRNA	1 658 683
miRNA–circRNA	1267
circRNA–protein	82
circRNA–mRNA	15
Others^a^	97 445

^a^RNA interactions of other categories (such as ‘snoRNA-pseudogene’, ‘snRNA-pseudogene’, etc).

**Table 3.** Statistics of RNA–DNA interactions

Technologies	Interactions
ChIRP-seq data	2 450 577
CHART-seq data	915 101
iMARGI, ChAR-seq and GRID-seq	4 963 704

Apart from basic information such as species, cell line and experimental methods, biomolecules and interactions were provided with more detailed annotation, including interaction level, interaction class, tags of interactions and PhastCons sequence conservation score of the binding site. Levels were defined as ‘RNA–protein’, ‘RNA–RNA’, ‘RNA–DNA’ and ‘RNA-TF’ based on the types of interacting molecules. According to the interacting classes, interacting pairs were classified as different types (such as ‘binding’, ‘regulatory’, etc). Tags including ‘miRNA target interaction’, ‘ncRNA–protein binding’ and others were applied to briefly describe the mechanism of each interaction with visualization displayed on the website. For each binding site detected in CLIP-seq, we provide an average PhastCons score to present evolutionary conservation. All the detail information of interactions can be viewed on the Interaction Profile page (http://bigdata.ibp.ac.cn/npinter5/browse/). For each interacting molecule, such as lncRNA, circRNA and miRNA, we re-annotated with NONCODE IDs, circBase IDs and miRBase IDs respectively. We arranged the type, description, alias, interaction tables and related disease annotation into the Molecule Profile page (e.g. http://bigdata.ibp.ac.cn/npinter5/molecule/22789/). Users can easily browse and view these data on our website.

We added ncRNA interactions from several new sources to NPInter v5.0, which greatly expanded our data volume. This is detailed as follows.

#### Global RNA–DNA interactions

Several studies have shown that ncRNA-chromatin interactions have impact on the transcription of genes. For example, *Drosophila* ncRNAs *roX1* and *roX2* are involved in dosage compensation (37) and the lncRNA, *HOTAIR*, recruits proteins by interacting with chromatin (38). We included ncRNA–DNA interactions derived from ChIRP-seq datasets in the previous version, and we newly integrated 915 101 pairs of RNA–DNA interactions from CHART-seq in NPInter v5.0 because this method is widely applied. However, both ChIRP and CHART technologies are limited to one RNA at a time, and lack a global view of all potential RNA-chromatin interactions (17). Recently, iMARGI, ChAR-seq and GRID-seq technologies have been developed and greatly increased the number of RNA–DNA interactions by their ability to globally localize chromatin-associated ncRNAs. This enables the depiction of a more comprehensive RNA–DNA network. We therefore integrated interactions from these three techniques into NPInter v5.0. A total of 8 329 382 RNA–DNA interaction pairs involving 16 806 ncRNAs (15 in NPInter v4.0) were collected, greatly expanding the data volume.

#### SARS-CoV-2 related RNA interactions

Severe acute respiratory syndrome coronavirus-2 (SARS-CoV-2) has caused large-scale outbreaks of coronavirus disease 2019 (COVID-19) all over the world. It is an RNA virus whose success as a pathogen relies on its abilities to repurpose host RBPs and to evade antiviral RBPs (39). Numerous interactions between the virus and host have been described, which may play essential roles in its evasion. For example, host cellular La-related protein1 (LARP1) binds genomic and subgenomic SARS-CoV-2 RNAs to repress SARS-CoV-2 replication in infected human cells (40), and the viral RBP, ORF9b, may contribute to SARS-CoV-2 infection by interacting with virus RNA (41). Also, the SARS-CoV-2 genome engages in different interactions with cellular host RNA (42). Multiple studies have captured thousands of RNA interactions related to SARS-CoV-2 (39–44). However, these data have not been integrated. To provide a comprehensive reference for SARS-CoV-2 research to find antiviral drug targets, we produced a dedicated SARS-CoV-2 RNA interaction collection including five panels for different types of interactions between virus and host. We manually extracted 1567 interactions from studies that employed RNA interactome capture technologies such as cRIC (45), RAP-MS (46) and ChIRP-MS (47). This is the first RNA interaction resource for COVID-19 and we will keep updating the newest findings in this area.

#### Interactions of lncRNAs with cell type-specific expression from scRNA-seq data

The tumor microenvironment (TME) is a highly heterogeneous and constantly evolving milieu composed of various cell types ranging from normal to malignant cells (48). These cell types perform different functions in the tumor. Cell type-specific gene expression helps to confer cellular functions and ncRNAs such as lncRNAs have distinct cellular functions in the TME (49). For example, pancreatic ductal adenocarcinoma (PDAC) cancer cells showed heterogeneity and were divided into four major subclusters, one of which marked by lncRNA *MEG3* was mostly contributed to metastatic PDAC (50). scRNA-seq was considered as an unprecedented technology to explore cell type-specific expression compared with bulk RNA-seq in which various cell types contribute to an averaged signal. Cell type-specific expression of lncRNAs such as *DLX6-AS1, LOC646329* and *H19* has been identified in scRNA-seq datasets (51,52). Here, we preferentially acquired cell type-specific lncRNAs from tumor scRNA-seq data and annotated lncRNAs with expression heterogeneity. After screening, 20 scRNA-seq datasets (including 883 330 cells) representing 20 cancer types were collected and processed to identify specifically expressed lncRNAs in different cell types, and their interaction pairs existing in our database were presented. Cell type distribution of each scRNA-seq dataset was displayed and 1422 lncRNAs from 248 cell types were identified. We extracted marker genes and functional pathways from corresponding studies to depict cell states for each cell type. Information of cell type-specific lncRNAs was provided in detail, and interaction pairs could be accessed in the Molecular Profile page. We believe that these interactions with cell specific molecules will contribute to the study of cancer.

### An example of database usage

**Figure 2.**
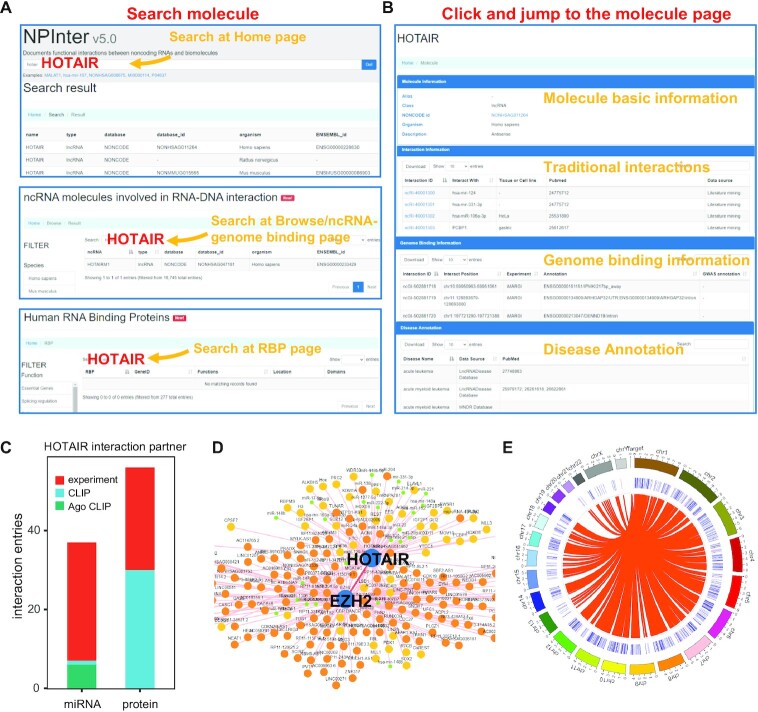
An example in NPinter v5.0. (**A**) Search lncRNA HOTAIR at Home page (top), Browse/ncRNA-genome bindings page (middle) and RBP page (bottom), respectively. (**B**) Click and jump to the molecule page of HOTAIR which contains the annotations of molecule basic information, traditional interactions, genome binding information and disease annotation. (**C**) Number of interaction entries of HOTAIR from different types and data sources. Experiment refers to low-throughput experiments such as dual-luciferase reporter gene assay, RNA pull-down assay, RNA immunoprecipitation assay, etc. (**D**) The lncRNA HOTAIR and protein EZH2 interaction network. (**E**) HOTAIR genome binding diagram in the molecule page.

Users can retrieve various types of interactions for a given molecule in NPInter. Here we take lncRNA *HOTAIR* as an example, which is one of the most extensively studied lncRNAs. Users could search *HOTAIR* at webpages of ‘Home’ and ‘Browse’. (Figure 2A). You can also search it at ‘RBP’ webpage but obtain no results (Figure 2A). Then click the human record on the search result and jump to the molecule page. This page contains molecule basic information, traditional interactions, DNA binding information and disease annotation for a specific molecule, which can all be downloaded (Figure 2B). 93 interaction records of *HOTAIR* were retrieved in the traditional interaction panel (Figure 2B). The interaction partners consisted of miRNAs and proteins, and a large portion of the entries was validated experimentally (Figure 2C). *HOTAIR* has been proved to directly bind polycomb repressive complex 2 (PRC2) to coordinate chromatin occupancy (53). The components of PRC2, including EZH2, SUZ12 and EED, are all collected as *HOTAIR* interaction partners in our database. There is an interaction profile page for every interaction record, such as interaction between *HOTAIR* and EZH2 (ncRI-40001312), in which the interaction network is displayed (Figure 2D). The DNA target sites of *HOTAIR* are listed in the genome binding information panel of the molecule page (Figure 2B). These target sites are visualized in the same page (Figure 2E). In summary, NPInter provides comprehensive annotations of interactions.

### Comparison with other databases

Currently, there are several databases for RNA interactions such as starBase (54), LncTarD (55), miRTarBase (56) and RNAInter (57). starBase dedicated to identify RNA–RNA and protein–RNA interactions from high-throughput sequencing data of CLIP-seq. LncTarD documented experimentally-supported functional lncRNA-target regulations in human diseases, and miRTarBase deposited interactions between miRNAs and their target genes from manually curated articles and CLIP-seq data. RNAInter has integrated all interaction data from NPInter v4.0. NPInter aims to arrange and provide comprehensive interactions for ncRNAs (such as lncRNAs, miRNAs and circRNAs, etc). Compared with these similar databases, NPInter provides more multidimensional interactions for ncRNAs (RNA–protein, RNA–RNA, RNA–DNA) (see Table 4). Also, our database contains both experimental validated RNA interactions by literature mining and data generated by high-throughput sequencing experiments with disease and functional annotation. Moreover, NPInter covers global RNA–DNA interactions and integrated SARS-CoV-2 RNA interactions, which other databases have not documented.

**Table 4.** Comparison with other databases

	starBase	LncTarD	miRTarBase	RNAInter	NPInter
**Types**					
RNA–protein	**√**	**√**		**√**	**√**
RNA–RNA	**√**	**√**	**√**	**√**	**√**
RNA–DNA				**√**	**√**
COVID-19					**√**
**Methods**					
Literature mining		**√**	**√**		**√**
CLIP-seq	**√**			**√**	**√**
Ago CLIP-seq	**√**		**√**	**√**	**√**
ChIRP-seq, CHART-seq				**√**	**√**
iMARGI, ChAR-seq, GRID-seq					**√**

We integrated abundant RNA interactions with experimental evidence from published literature (18 181 interactions from 7311 articles) and applied strict *P* value thresholds in reliable softwares (such as macs2 and piranha) when processing high-throughput data. In summary, NPInter is dedicated to providing multidimensional interactions for each ncRNA while maintaining high data quality.

### Service update

We updated existing modules and rearranged our web interface to incorporate the new modules mentioned above for better user interaction and experience. The Browse module was updated to be categorized as traditional interactions and ncRNA-genome bindings. The former contains interactions of RNA–protein, RNA–RNA and RNA-TF derived from the existing Browse module, while the latter deposits chromatin-associated ncRNAs. The SARS-CoV-2 module was built to exhibit SARS-CoV-2 related interactions and annotations of each biomolecule. Also, the scNPInter module was developed for data visualization and display of cell type-specific lncRNAs and their interaction pairs from scRNA-seq datasets. Additionally, we integrated a new module named RBP to provide the interactions of RNA-binding proteins with corresponding annotations.

#### RBP module

RBPs are proteins that bind to double or single stranded RNA to play essential roles in RNA-mediated gene regulation (58). The regulatory functions of RBPs are critical for normal human physiology because defects in RBP function are associated with diverse genetic and somatic disorders, such as neurodegeneration, auto-immune defects, and cancer (59). Many researchers are interested in the interactions between RBPs and their targets, as can be seen from the user feedback of NPInter v4.0. For NPInter v5.0, we built a new RBP module for browse. To work towards exhibiting more detailed binding and function of the human RBPs we collected, we annotated 277 RBPs with localization, RNA binding domains, and function from ENCODE project phase III (60). Interactions between a certain RBP and its targets can be easily browsed and downloaded on our website. Users can filter RBPs with different properties. We hope that this module will provide a useful and convenient resource portal for the study of the RNA-RBP network.

## CONCLUSION

Unlike protein-protein interactions, which have been extensively investigated, ncRNA interactions have received less attention and remain largely unknown. To well document the ncRNA interactions and make a comprehensive complement to the biological regulatory network, we constructed the NPInter database and have updated it to a fifth version. In NPInter v5.0, we doubled the number of ncRNA interactions primarily by literature mining and high-throughput data processing. The volume of experimentally validated ncRNA interactions has been greatly expanded from the large accumulation of recently published literature. Each interaction pair was annotated with detailed information and conservation scores, and each molecule was annotated with disease information. In addition to ChIRP-seq and newly added CHART-seq which can only target one chromatin-associated ncRNA, we have integrated global RNA–DNA interactions derived from iMARGI, ChAR-seq and GRID-seq technologies. This has greatly increased the number of RNA–DNA interactions and chromatin-associated ncRNAs. To provide reference for the study of COVID-19, we also collected different types of SARS-CoV-2–RNA interactions between virus and host. Specificity of lncRNA expression in different cell types from tumor scRNA-seq data were also integrated in this version to provide a cell-type level view of interactions. Based on user feedback, a new RBP module was presented to provide RBP-based interactions classified according to annotation of localization, binding domains and functions. Compared with other RNA interaction databases, NPInter v5.0 covers global RNA–DNA interactions and integrated SARS-CoV-2–RNA interactions with functional annotations.

The study of RNA interaction has expanded into various areas, such as tumor biology and COVID-19 study. Moreover, new technologies for identifying interactions are constantly being developed. We will continuously update and maintain the NPInter database to document such interactions. There are several techniques that investigate RNA interaction directly in a small number of cells or at single cell level, such as LACE-seq (61). Detecting RNA interactions at the cellular level is becoming more and more feasible, although we can only identify cell type-specific lncRNAs and show related interactions due to current limitation of technology. We will keep updating our scNPInter section when these single cell related techniques are developed and widely applied. Furthermore, NPInter is a member of our systematic platform for noncoding RNAs, which provides databases and webtools for analysis of ncRNAs, including NONCODE (23), piRBase (62) and smProt (63). Consequently, we hope that NPInter will provide useful information of RNA interactions and be a valuable web service for the scientific community.

## DATA AVAILABILITY

NPInter v5.0 is free to access, browse, search and download at http://bigdata.ibp.ac.cn/npinter5.

## Supplementary Material

gkac1002_Supplemental_File
